# RNA-Seq based genetic variant discovery provides new insights into controlling fat deposition in the tail of sheep

**DOI:** 10.1038/s41598-020-70527-8

**Published:** 2020-08-11

**Authors:** Mohammad Reza Bakhtiarizadeh, Ali A. Alamouti

**Affiliations:** Department of Animal and Poultry Science, College of Aburaihan, University of Tehran, Tehran, Iran

**Keywords:** Animal breeding, Genomics, Sequencing

## Abstract

Genetic basis of fat deposition in sheep tail have not been completely elucidated yet. Understanding the genetic mechanisms controlling fat-tail size can improve breeding strategies to modulate fat deposition. RNA sequencing has made it possible to discover genetic variants that may underlie various phenotypic differences. Hence, to identify genetic variants that are important for describing different fat-tail phenotypes in sheep, RNA sequencing was used for single nucleotide polymorphism (SNP) calling in two Iranian sheep breeds (Lori-Bakhtiari, fat-tailed; n = 4, vs Zel, thin-tailed; n = 4). Using a stringent pipeline, a total of 112,344 known SNPs were genotyped, of which 30,550 and 42,906 SNPs were shared by at least two Lori-Bakhtiari and Zel, respectively. Comparing these SNPs showed 2,774 (including 209 missense and 25 deleterious SNPs) and 10,470 (including 1,054 missense and 116 deleterious SNPs) breed-specific SNPs in Lori-Bakhtiari and Zel sheep, respectively. Potential breed-specific SNPs were detected by considering those located in QTL regions associated with fatness or reported as important candidates in previous similar studies. Of the breed-specific SNPs, 724 and 2,905 were located in the QTL regions. Functional enrichment analysis of the affected genes revealed several enriched gene ontologies and KEGG pathways related to fat metabolism. Based on the results, several affected genes were proposed to be strongly linked with fat deposition such as DGAT2, ACSL1, ACACA, ADIPOQ, ACLY, FASN, CPT2, SCD, ADCY6, PER3, CSF1R, SLC22A4, GFPT1, CDS2, BMP6, ACSS2, ELOVL6, HOXA10 and FABP4. Moreover, several SNPs were found in the candidate genes related to fatty acid oxidation introducing them as promising candidates responsible for lower fat content in tail of Zel. Our findings provided new insights into the genetic mechanisms of fat deposition in sheep, which can serve to designing appropriate breeding programs.

## Introduction

Sheep is recognized as the first domesticated grazing animal. It is believed that humans began mediating the breeding of sheep 9,000–11,000 years ago (end of the Mesolithic period), when sheep were selected for production of meat, wool, milk, etc.^1^. As many as 1,400 recorded breeds of sheep each with its own distinctive morphology^2^ have been developed as a consequence of evolutionary responses to a wide variety of geographical and sociocultural environments. Of these population, more than one-fourth are fat-tailed restoring a large amount of energy as fat mass in the tail^3^. From the evolutionary perspective, fat-tailed sheep was originally branched from thin-tailed breeds reflecting the need for a critical energy store in harsh environments^4^. However, todays’ intensive production systems criticize fat-tailed breeds because fat deposition requires a greater energy cost than accretion of an equivalent amount of lean tissue. Moreover, fat-tail comprises as much as 20% of carcass weight, which drastically decreases the economic value of the carcass^3^. Therefore, sheep breeders need to identify the mechanisms that genetically control tail fat development in order to design breeding programs for reducing tail size.

To date, various genomic approaches such as genome-wide association study, selection signature studies or gene expression analysis have been used to characterize the potential genetic background of fat deposition in a variety of fat-tailed sheep breeds. Based on a genome-wide detection of selective signatures study, Moioli et al. reported two genes (BMP2 and VRTN) as potential candidates related to the fat-tailed phenotype of sheep^5^. Yuan et al. compared fat‐tailed and thin‐tailed Chinese sheep breeds using genome-wide selective signature analysis and identified 6.24 Mb candidate regions and some genes (PPP1CC, SP3, HOXA11, WDR92, BMP2, SP9, PROKR1 and ETAA1) that can be related to fat-tail formation^6^. HOX and HOX-related genes were proposed as important genes associated with fat-tail development in a fat-tailed sheep breed (Tan)^7^. In a recent study, transcriptome of samples from longissimus dorsi muscle, perinephric fat and fat-tail of Lanzhou sheep, were subjected to RNA-Seq and 606,952 SNPs were identified overlapping with QTLs of fat traits. The latter study also suggested three potential genes (CREB1, WDR92 and ETAA1) related to fat-tail development^8^. Also, whole genome sequencing identified T gene as an important candidate gene influencing tail size in Hulunbuir short-tailed sheep^9^. Nonetheless, limited studies have focused on comparing the two extremely different breeds of Iran origin, i.e. Lori-Bakhtiari and Zel. To clarify, these are two of over 28 Iranian sheep breeds^3,10^ differing in many morphological features and phenotypes. Lori-Bakhtiari is one of the heaviest breeds in the south-western regions of Iran with a large fat-tail often hanging down the hocks. In the opposite, Zel is the only thin-tailed Iranian sheep with a small frame mainly distributed in the northern ranges of the Alborz mountain around the Caspian Sea^3,10,11^. In one study, three regions located on chromosomes 5, 7 and X were reported to link with fat-tail development in Lori-Bakhtiari^12^. Additionally, in our previous studies, different mRNA and lncRNA genes with potential roles in fat-tail development were characterized in these breeds^3,10,11^. These studies have provided a basis for understanding the genetic mechanisms underlying fat deposition as well as genetic diversity between these breeds. However, to the best of our knowledge, there is no study comparing the genetic variants of these breeds by high-throughput sequencing methods. Moreover, molecular genetic basis related to fat-tail development has not been completely elucidated and further studies are required to explore these aspects. It is well known that natural and artificial selection leaves detectable signatures on the genome^13^. Hence, a deserving aspect is to research for genetic variant differences between thin and fat-tailed sheep, such as Lori-Bakhtiari and Zel, which may affect fat deposition. Particularly, the obvious phenotypic differences of these breeds make them an ideal model to identify potential genetic variants associated with fat deposition.

High-throughput RNA sequencing (RNA-Seq) analysis has been extensively used for gene expression profiling^3^. Besides, RNA-Seq is a powerful tool for identifying alternative splicing^14^, long non-coding RNAs^10,15^, allele specific expression^16^, RNA editing^17,18^ and genetic variant discovery. The usefulness of RNA-Seq for efficient single nucleotide polymorphism (SNP) discovery in transcribed genes has been demonstrated in different tissues and species^19–21^. Compared to DNA sequencing, SNPs calling analysis based on RNA-Seq data is remarkably cost-effective and yields almost 100% efficiency^20,22^. Furthermore, most SNPs identified via this analysis are located in the transcribed regions of the genome, wherein variants are most likely to result in phenotypic changes and undergo selection pressure^23^. Therefore, RNA-Seq method was used to identify gene-based SNPs and to investigate if such SNPs are associated with differences in tail phenotype between Lori-Bakhtiari and Zel breeds.

## Material and methods

### Animals and samples

**Figure 1:**
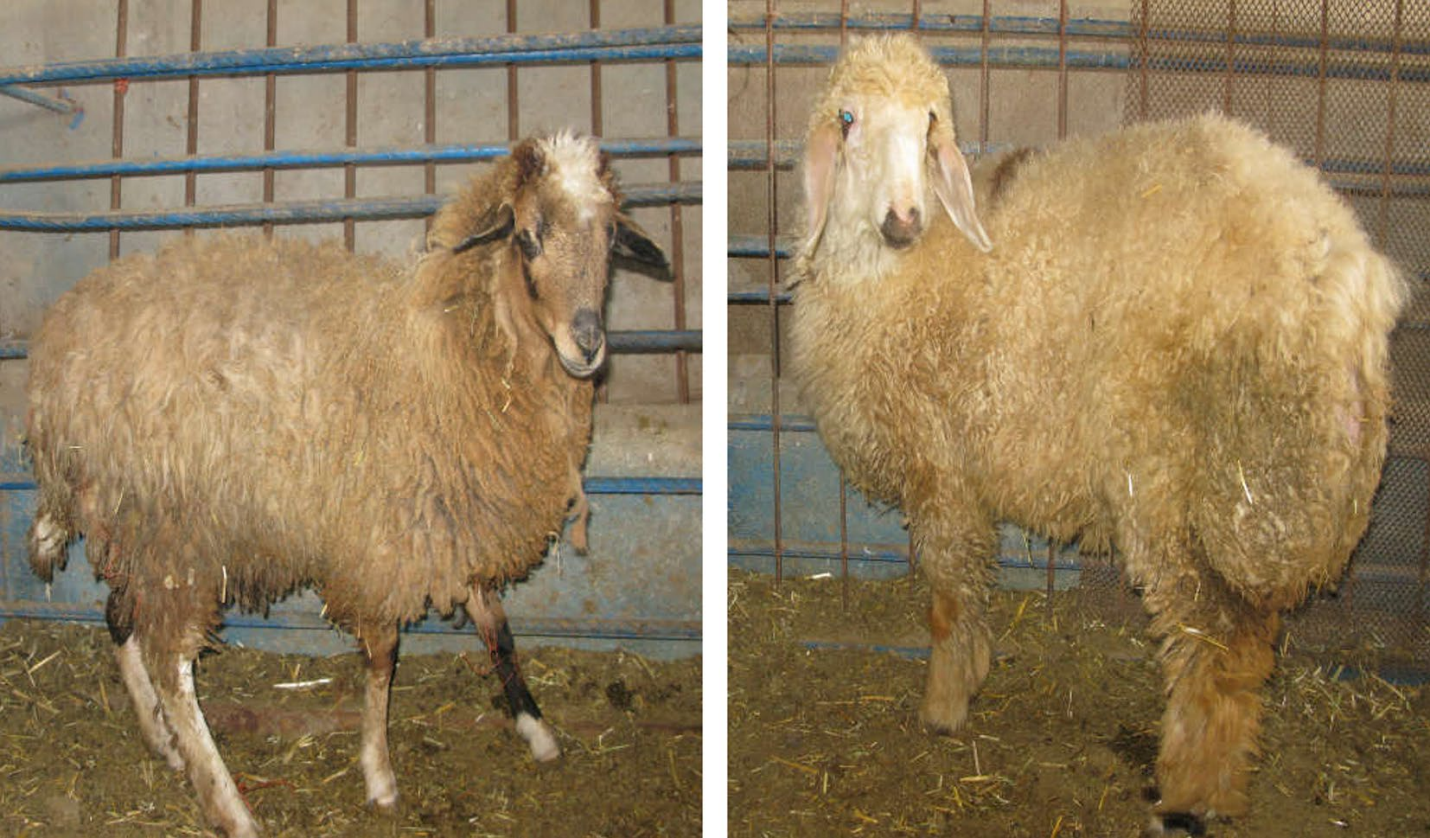
Examples of Zel (thin-tailed breed, left) and Lori-Bakhtiari (fat-tailed breed, right).

RNA-Seq dataset used in this study was obtained from our previous study along with two new samples (one from Lori-Bakhtiari and one from Zel, Fig. 1), all of which were treated with the same protocols. Detailed information about the samples and the experimental design have been described previously^3^. Briefly, fat-tail tissues of the sheep were collected immediately after the animals were slaughtered at seven months of age (four Lori-Bakhtiari and four Zel sheep). These lambs were housed for 120 days in individual pens (at the research station of the college of Aburaihan, University of Tehran) under the same environmental and dietary conditions with ad libitum access to the diet and water. All tissue samples were snap frozen in liquid nitrogen and then transferred to a − 80 °C freezer until required for RNA isolation.

### RNA isolation and sequencing

The total RNA was extracted and prepared from 100 mg of fat taken from tail tissues using the Tripure isolation reagent kits (Roche Applied Science); detailed procedures are described in our previous study^3^. In total, eight samples comprised of fat-tail tissues of four Lori-Bakhtiari and four Zel sheep breeds. The quantity and quality of samples were evaluated using the NanoDrop (Thermo Scientific NanoDrop 2000) and 1% agarose gel electrophoresis, respectively. Then, high quality samples (28S/18S > 1.8 and OD 260/280 ratio > 1.9) were sent to BGI company (Shenzhen, China) to construct cDNA library and RNA sequencing. Samples were applied for sequencing if the RNA Integrity Number (RIN) was > 7, based on an Agilent Bio Analyzer 2,100 system. The sequencing was performed on an Illumina HiSeq 2000 platform by paired-end strategy (read length 150 bp). The dataset is available in SRA database, under BioProject accession number PRJNA508203.

### RNA-Seq data processing

RNA-seq reads were processed through a quality control and trimming approach using FastQC (v0.11.5)^24^ and Trimmomatic (v0.35)^25^ tools to remove low-quality reads/bases and adaptor sequences, respectively. Trimmomatic was executed using its adaptive trimming algorithm, maximum information (MAXINFO:120:0.9), to balance the benefits of retaining longer reads against the drawback of having low-quality bases. Also, minimum phred score (TRAILING) and minimum length (MINLEN) were set to 20 and 120, respectively.

### SNPs calling and annotation

The clean reads were used for SNP calling and insertions and deletions (indels) were ignored, since accurate indel calling is still challenging to implement^26^. To do this, a comprehensive and stringent filtering pipeline was applied to distinguish putative SNPs from sequencing errors, computational mapping errors, RNA editing and other errors as follow:

1. The clean reads were aligned to the Ensembl ovine reference genome (Oar_v3.1, GCA_000298735.1) using Hisat2 software (v2.1.0)^27^. A list of exon-exon junctions extracted from the known gene model annotation (Ensembl release 94) was used to guide the read mapping.
2. Only uniquely and concordantly paired-end mapped reads were kept to minimize the detection of false-positive SNPs.
3. Identical reads (or PCR duplicates) that were aligned to the same location, were marked using MarkDuplicates tool from Picard (v1.104(1627)) (https://picard.sourceforge.net/) and were ignored in downstream analysis.
4. To correct the misalignment due to higher alignment errors around indels, regions containing indels were called using RealignerTargetCreator from Genome Analysis Toolkit (GATK) tool (v3.5) with the knowledge of where variants are likely to be, based on Ensembl ovine SNP database. Then, reads were realigned by IndelRealigner from GATK tool (v3.5)^21^.
5. BaseRecalibration function of GATK was applied to recalibrate the base quality scores, supplying lists of Ensembl ovine SNP database, to solve the problems of over- or under-estimated scores during sequencing processes.
6. GATK's HaplotypeCaller function was used to call variants with a stand_call_conf and stand_emit_conf value of 30 and mbq of 25.
7. Initial list of identified variants was filtered according to a number of standard quality metrics (Total depth of coverage < 10, HomopolymerRun > 5, RMSMappingQuality < 40, MappingQualityRankSum < -12.5, QualitybyDepth < 2 and ReadPosRankSum < -8). These variant annotation cut‐offs led to eliminate the variants with distance bias, mapping quality bias, homopolymer bias and sites with less than 10 supporting reads.
8. To obtain high-confidence SNPs, the variants were further filtered as follow: keep the variants with only one nonreference type, discard the variants with less than three reads supporting the SNP and remove the variants that were located in a) regions with bidirectional transcription, b) simple sequence repeats regions (± 3 bases) and c) splice junctions regions (within 5 bp intronic flanking).
9. The remaining variants were retained for downstream analysis, if they were reported as known SNPs in Ensembl ovine SNP database.
10. Principal component analysis (PCA) was applied by SMARTPCA method^28^ as implemented in the R package SNPRelate^29^.
11. Since, our main goal was to characterize SNPs that may regulate fat-tail development, identified SNPs of the two sheep breeds, were compared. First, SNPs that were common to at least two animals in each breed, were considered common-SNPs. Then, common-SNPs with divergent genotypes between Lori-Bakhtiari and Zel (homozygous or heterozygous in one breed and alternative allele homozygous in the other) were considered breed-specific SNPs per given breed. In other words, those SNPs segregating only in one breed were used for downstream analysis.
12. Ensembl’s Variant Effect Predictor tool (VEP, v97.0)^30^ was used to functionally annotate the impact of the identified SNPs as well as their locations in the genes. Also, sorting intolerant from tolerant (SIFT) algorithm of VEP tool was applied to report the effect of missense single amino acid substitutions. SIFT is a sequence homology-based algorithm and predict whether a SNP is tolerated or not tolerated (SIFT score ≤ 0.05) based on the degree of evolutionary conservation among homologous proteins across multiple species^31^.

### QTL analysis

To analyze if any of the identified SNPs were genetically associated with fat metabolism, a colocalization analysis was performed with fat-associated QTLs. All the sheep QTLs associated with fat metabolism obtained from AnimalQTLdb (Release 38)^34^. The current version of the sheep QTLdb includes 3,001 QTLs representing 256 different traits. Of these, 159 QTLs associated with 18 traits related to fatness were considered such as “Abdominal fat weight”, “Carcass fat percentage”, “Fat density”, “Intermuscular fat weight”, “Subcutaneous fat area”, “Subcutaneous fat thickness”, “Subcutaneous fat weight”, “Total fat area” and “Tail fat deposition”. Then, breed-specific SNP positions for each breed were compared with QTLs locations.

### Functional enrichment analysis

Gene ontology (GO) and KEGG pathway enrichment analysis of the genes containing breed-specific SNPs was performed using EnrichR^35^ to explore the potential involvement of the affected genes in biological processes related to fat metabolism. Those GO terms or KEGG pathways showing adjusted P-value (based on false discovery rate, FDR) less than 0.05 were considered significant.

### Gene expression analysis

StringTie software (v1.3.4d)^32^ supplied by the Ensembl annotation (release 94) was used to perform a reference annotation-based transcript assembly for each sample. To account for the differences in the library sizes, abundances of transcripts were upper-quartile normalized. Moreover, the genome reference sequence was supplied for sequence specific bias correction in order to improve the expression estimates. The expression values of all the identified transcripts were calculated as FPKM (fragments per kilobase per million) and Cuffdiff tool (v2.2.1)^33^ was used to identify differentially expressed genes (DEGs) across the two breeds using a beta negative binomial model. Genes with a false discovery rate (FDR) ≤ 0.05 were considered as DEGs.

### Ethics statement

All methods were carried out in accordance with relevant guidelines and regulations. All experimental protocols were approved by a research council of the University of Tehran.

## Results

### Sequencing and alignment

**Figure 2:**
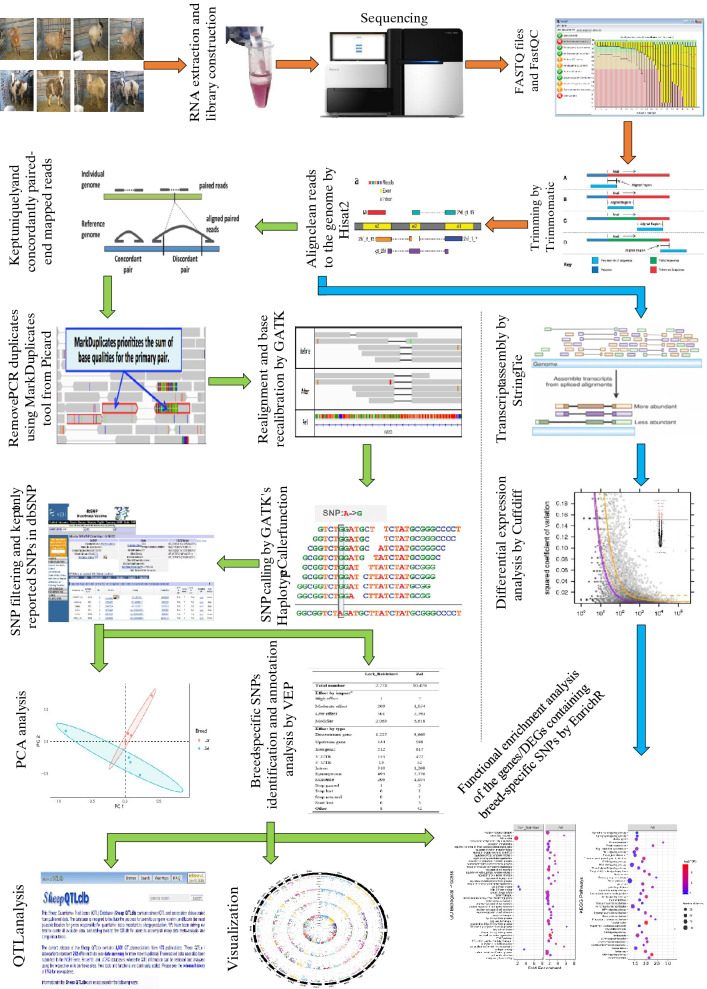
The bioinformatic pipeline for SNP calling, differential expression analysis and functional enrichment analysis.

The stringent stepwise filtering pipeline was used for SNP calling, differential expression analysis and functional enrichment analysis (Fig. 2). Approximately, 175 million paired-end reads were generated from RNA sequencing of the eight fat-tail tissues, an average of ~ 22 million paired-end reads per sample. After quality-check of raw reads, only 1,570 reads were removed demonstrating high quality of the RNA-Seq data. On average, 83.27% of the clean reads in each sample were aligned with sheep genome. Also, mean percentage of the reads mapped concordantly and uniquely to the genome was 69.12% (Table 1).

**Table 1:** Descriptive statistics of RNA-Seq data and alignment (number (%)).

Samples	Raw reads	Trimmed reads	Total mapping	Uniquely and concordantly mapped
Lori_Bakhtiari_1	26,282,890	26,282,599	23,596,517 (89.78)	20,652,003 (78.58)
Lori_Bakhtiari_2	20,075,866	20,075,798	15,466,394 (77.04)	12,798,432 (63.75)
Lori_Bakhtiari_3	20,428,424	20,428,247	15,572,452 (76.23)	12,591,179 (61.64)
Lori_Bakhtiari_4	18,061,231	18,061,047	16,009,312 (88.64)	13,402,952 (74.21)
Zel_1	22,292,871	22,292,640	19,240,777 (86.31)	15,964,723 (71.61)
Zel_2	20,164,542	20,164,351	15,486,221 (76.8)	12,547,998 (62.23)
Zel_3	20,532,170	20,532,024	16,542,651 (80.57)	13,214,997 (64.36)
Zel_4	26,926,569	26,926,287	24,459,839 (90.84)	20,622,189 (76.59)

### SNP detection and functional annotation

**Figure 3:**
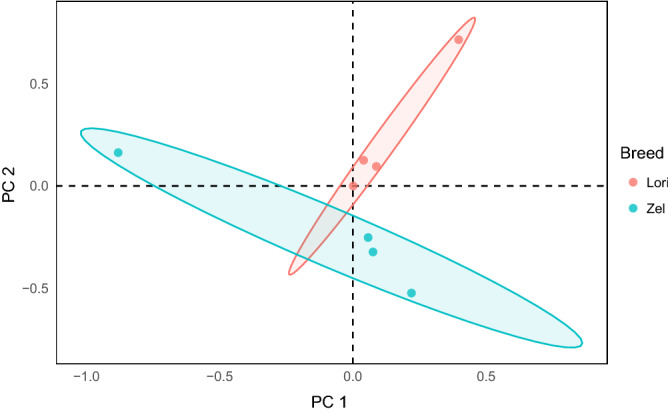
PCA plot of SNP genotypes in the sheep breeds.

The GATK pipeline resulted in 133,051 and 147,422 variants presented specifically in Lori-Bakhtiari and Zel, respectively. Totally, 120,049 confident SNPs were detected in all analyzed samples after variant filtering. Of these, 112,344 (~ 94%) SNPs were annotated as known SNPs in Ensembl ovine SNP database and were considered for further analysis. Results of PCA analysis based on the first two principal components showed that the eight samples can be divided into two clusters according to their breed (Fig. 3).

**Figure 4:**
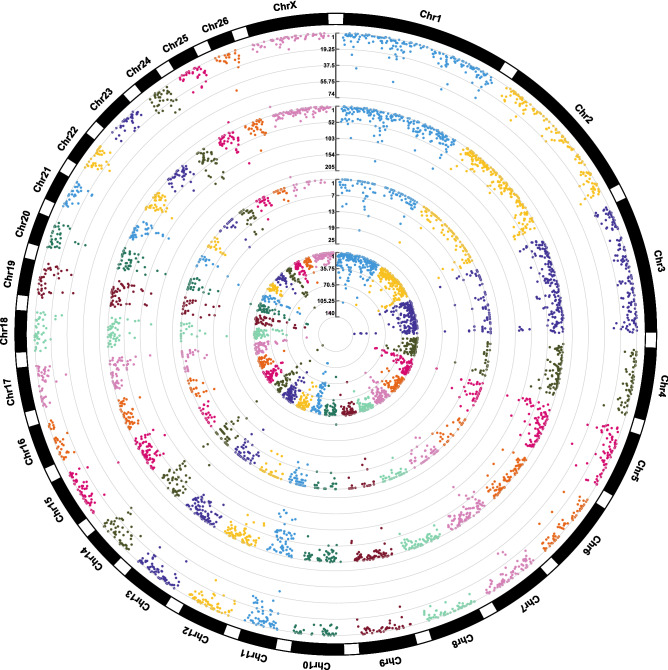
Circos plot of distribution and densities of common and breed-specific SNPs in Lori-Bakhtiari and Zel breeds. The outer layer shows the chromosomes. The common and Zel specific SNPs are positioned in the first and second inner layers, respectively. The common and Lori-Bakhtiari specific SNPs are positioned in the third and fourth inner layers, respectively. Vertical lines in each layer represent the number of SNPs in that position.

Out of 112,344 identified SNPs, 68,089 and 93,846 SNPs were observed in Lori-Bakhtiari and Zel breeds, respectively. The transition to transversion ratio (Ts/Tv) was similar for the two breeds (Lori-Bakhtiari:2.95 and Zel:2.98). On average, the Ts/Tv was 2.97 (2.91–3.05) across all samples showing a high ratio compared to all reported SNPs in dbSNP database (2.43). Typically, Ts/Tv is higher for SNPs in exons and coding sequences, which is attributed to the increased presence of methylated cytosine in CpG dinucleotides in these regions. Moreover, a higher Ts/Tv usually indicates higher accuracy of variant calling^36^. The rate of heterozygous/homozygous SNPs was 0.54% (0.45–0.56) across all samples (Lori-Bakhtiari:0.53 and Zel:0.55). A total of 30,550 SNPs was shared by at least two Lori-Bakhtiari (Lori-Bakhtiari common-SNPs) and a total of 42,906 SNPs was shared among Zel (Zel common-SNPs). Comparison of common-SNPs of the two breeds showed 2,774 and 10,470 breed-specific SNPs in Lori-Bakhtiari and Zel, respectively (Supplementary File S1). SNP density across the genome showed almost a uniform distribution in both breed-specific SNPs (Fig. 4).

Functional prediction consequences of the breed-specific SNPs are summarized in Table 1. Results showed that impact pattern of the SNPs was similar for both breeds. In this way, high impact SNPs were much less frequent than modifier, moderate or low impact SNPs. Also, a similar pattern of SNP locations on genome was found for both breeds (Table 2, Supplementary File S1). Out of 705 Lori-Bakhtiari specific exonic SNPs, 209 were missense substitution (non-synonymous), 25 of which were predicted as damaging variants (or deleterious). Annotation analysis of 3,830 Zel specific exonic SNPs identified 1,054 missense SNPs, of which 116 were predicted as being deleterious (Table 2, Supplementary File S1).

**Table 2:** Characteristics of the identified breed-specific SNPs in Lori-Bakhtiari and Zel breeds.

	Lori_Bakhtiari	Zel
Total number	2,774	10,470
Effect by impact*		
High effect	1	7
Moderate effect	209	1,054
Low effect	501	2,791
Modifier	2,063	6,618
**Effect by type**		
Downstream gene	1.227	3,660
Upstream gene	144	581
Intergenic	212	617
3′-UTR	155	472
5′-UTR	13	52
Intron	310	1,208
Synonymous	495	2,776
Missense	209	1,054
Stop gained	1	3
Stop lost	0	1
Stop retained	0	1
Start lost	0	3
Other	8	42

*High effect the SNP is assumed to have disruptive impact in the protein, *Moderate effect* A non-disruptive SNP that might change protein effectiveness, *Low effect* assumed to be mostly harmless or unlikely to change protein behavior, *Modifier* usually non-coding SNPs or SNPs affecting non-coding genes.

### QTL analysis

By locating Lori-Bakhtiari specific SNPs in fatness related QTL regions, 724 (26%) SNPs were found in 1,155 QTL positions including 48 SNPs in “Total fat area” and 32 SNPs in “Tail fat deposition” QTLs. Among the 10,470 Zel specific SNPs, 2,905 (~ 28%) SNPs were located in 4,379 QTL positions including 161 SNPs in “Total fat area” QTLs and 128 SNPs in “Tail fat deposition” QTLs (Supplementary File S1).

### Functional enrichment analysis

**Figure 5:**
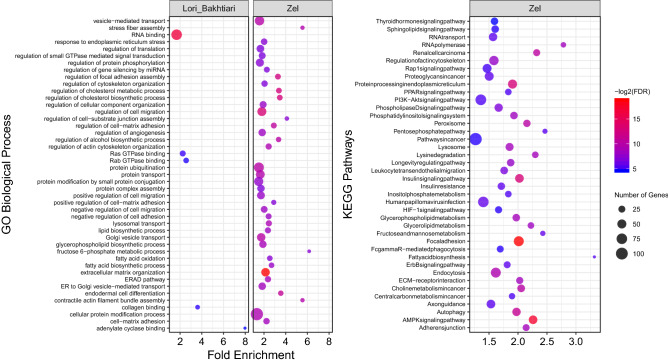
Functional enrichment analysis results. Because of the large number of significant GO terms (biological process) and KEGG pathways found in Zel breed, only the top 40 significant terms are displayed. Size and color of points represent -Log2 of FDR and number of genes associated with each term, respectively.

Lori-Bakhtiari specific SNPs were located in 1,253 coding genes that were significantly (FDR < 0.05) enriched in six biological process (BP) and five molecular function (MF) categories, but no significant KEGG pathway was identified. In this regard, 91 BP, nine MF and 67 KEGG pathways were significant with P-value < 0.05 and FDR < 0.3. They included fatty acid oxidation, fatty acid catabolic process, regulation of lipid biosynthetic process, MAP kinase activity, fatty acid degradation, etc. (Supplementary File S2, Fig. 5). On the other side, 3,244 genes containing Zel specific SNPs were significantly (FDR < 0.05) enriched in 168 BP, 49 MF and 39 KEGG pathways. Fatty acid biosynthetic process, fatty acid oxidation, fatty acid biosynthesis, fatty-acyl-CoA biosynthetic process and fatty acid synthase activity were examples of the significantly enriched GO terms and KEGG pathways. Also, 549 BP, 96 MF and 38 KEGG pathways were significant, with P-value < 0.05 and FDR < 0.3 including fatty acid transport, long-chain fatty-acyl-CoA biosynthetic process, regulation of fatty acid biosynthetic process, lipid particle organization, lipid homeostasis, phospholipid translocation, intracellular lipid transport, regulation of lipolysis in adipocytes, fatty acid degradation, fatty acid elongation, etc. (Supplementary File S3, Fig. 5).

### Gene expression analysis

In total, 306 DEGs were found, out of which 118 were up-regulated while 188 were down-regulated in Lori-Bakhtiari compared to Zel. The complete list of the identified DEGs are provided in Supplementary File S4. Out of 118 and 188 DEGs, 43 and 128 genes were respectively found that contained breed-specific SNPs. Twenty-two and 35 up and down-regulated genes containing breed-specific SNPs were found in Lori-Bakhtiari, of which nine up-regulated- and seven down-regulated genes were located in QTL regions associated with fat metabolism. Of these, RIMS3 containing a deleterious SNP was considered an important gene (Supplementary File S1). Likewise, 23 and 118 up and down-regulated genes containing breed-specific SNPs were found in Zel breed, of which seven up-regulated- and 42 down-regulated genes were located in QTL regions associated with fat metabolism. Also, two genes, namely COPA and MTOR, harboring deleterious SNPs were found (Supplementary File S1).

## Discussion

To identify the potential contribution of genetic variants to fat-tail development, SNP profile of two Iranian sheep breeds with extremely different tail shape (fat- and thin-tail) were compared. The accuracy of variant called by RNA-Seq data depends on the strength of filtering criteria set on variants^20,22^, therefore, a stringent filtering pipeline was applied to avoid possible errors during sequencing and computational analysis. Approximately, 6% of the identified SNPs (7,705 SNPs) were novel, not been previously annotated in Ensembl ovine SNP database. This result strongly suggests that genetic diversity of sheep remains to be investigated. Also, a higher number of SNPs that identified in Zel (93,846 vs. 68,089 in Lori-Bakhtiari) likely reflected higher genetic variation within Zel breed and its phylogenetic distance from the reference genome. Here, we focused on known SNPs, to maximize reliable SNPs and remove potential RNA editing sites^17,18^. PCA analysis based on 112,344 quality‐filtered SNPs demonstrated that samples from the same breed cluster together and can be separated by breed. In fact, samples from one breed cluster had distinctive genetic architecture compared to other.

In this study, only breed-specific SNPs were considered for down-stream analysis to provide a list of functional SNPs. The breed-specific SNPs are assumed to be genetic variants that are important for phenotypic differences between breeds, especially in the tail. However, fat-tail phenotype appears to be a complex phenomenon^37^ and some of the identified SNPs may also be related to other phenotypes than fat-tail formation. Hence, to strengthen the results, we focused on the SNPs/genomic regions/genes that were located in QTL regions associated with fat metabolism or reported as important candidates in fat-tail metabolism in the previous similar studies. More than 60% of the SNPs were located in non-genic section of the genome (down- and upstream gene, intergenic and intron), which can be attributed to the incomplete annotation of sheep genome while might be functionally transcribed genomic regions. The vast majority of the breed-specific SNPs were predicted to have modifier impacts as most of these SNPs were located in downstream gene regions.

The genomic position of the SNPs in QTL regions can help determine their putative functions. A total of 3,629 breed-specific SNPs was detected within the regions harboring sheep QTLs for fatness. In total, 160 SNPs were found in “Tail fat deposition” QTLs, all of which also located in the reported candidate regions associated with fat-tail development in a previous study^6^. Out of 25 deleterious SNPs in Lori-Bakhtiari, five SNPs were located in QTL regions related to fatness, two of which previously reported as candidate genes in tail fat formation^6^. They included a SNP (rs1094653489 in “Tail fat deposition” QTL) in METAP1D gene and another SNP (rs593140641 in “Carcass fat percentage” QTL) in RIMS3 gene. The RIMS3 gene was detected as an up-regulated gene in Lori-Bakhtiari, which makes it an interesting candidate for a follow-on experiment to assess its impact on fat-tail development. Also, one SNP (rs591141468 in “Subcutaneous fat area” QTL) was found in AMACR gene. AMACR is a mitochondrial and peroxisomal enzyme involved in betaoxidation of branched fatty acids and is well known as a fat metabolizer^38^ (Supplementary File S1).

In Zel breed, 32 of 116 deleterious SNPs were located in QTL regions associated with fatness. Among 32 SNPs, three were reported as candidate genes in fat-tail formation^6^ and were located in “Tail fat deposition” QTL regions including rs593851108 (in DYSF gene), rs429525525 (in CDHR1 gene) and rs161511466 (in ENSOARG00000008829). Moreover, nine coding genes harboring deleterious SNPs were identified in Zel with important roles in fat metabolism (ADIPOQ, MECR, TYSND1, EIF6, CSF1R, SYNJ1, MTOR, CSF1R and SLC22A4), elucidating their role in shaping fat-tail. For example, protein encoded by MECR is an oxidoreductase that catalyzes the last step in mitochondrial fatty acid synthesis type ɪɪ and overexpression of this gene increases peroxisome proliferator-activated receptor α (PPARα) activity^39^. The latter is actively involved in fatty acid catabolism during energy deprivation and is a key component of metabolic pathways initiating ketogenesis that, in turn, utilizing end products of fatty acid catabolism as major precursor. EIF6 is essential for lipid synthesis and increases the translation of transcription factors necessary for lipogenesis^40^. SLC22A4 was reported to have an association with fat-tail dimensions in sheep^42^. MTOR is a major mediator of lipid metabolism, which regulates lipogenesis, lipolysis and adipogenesis^41^. MTOR was found as an up-regulated gene in Zel that makes it a promising candidate for further evaluation. In Zel, four SNPs led to the creation of premature stop codons in ZNF862, PER3, ENSOARG00000012971 and ENSOARG00000008144 genes, which may shorten the proteins and affect their functions. These genes also harbored missense SNPs. Likewise, one Lori-Bakhtiari specific SNP caused a premature stop codon in ACD gene. Owing to the deleterious impact of the SNPs, these genes can be considered as potential candidate genes modulating fat deposition. In support of this, PER3 was shown to act as an inhibitor of adipocyte cell through inhibiting the transcriptional activity of PPAR so that PER3 knock-down mice showed greater fat content compared to wild-types^43^.

To investigate if the identified breed-specific SNPs were reported in other fat-tail sheep breeds, our results were compared with the previous cohort studies. Previous studies have reported inconsistent results, likely due to the statistical methodology, different breeds used or the complexity of fat-tail phenotype. However, under the hypothesis that the important genes/genomic regions related to fat-tail formation are evolutionarily conserved among different sheep breeds, we took into account the common SNPs/genes or genomic regions with previous similar studies. In total, 300 SNPs (78 SNPs in Lori-Bakhtiari and 222 SNPs in Zel) were concordant with previous studies on sheep breeds^6,8,44^. These SNPs were located within 91 genes (Supplementary File S1). Thirty one SNPs in Zel were detected in the seven candidate regions identified as having a relationship with fat-tail development in the study of Moradi et al. ^44^. Out of these, five SNPs were observed in ADCY6 gene, which were also located in “Internal fat amount” QTL region and therefore likely connected with fat deposition. In addition, 60 and 145 SNPs that were located, respectively, in 23 genes in Lori-Bakhtiari and 52 genes in Zel were also detected in candidate regions reported in Yuan et al. ^6^. Of these, for example, GFPT1 gene with five SNPs can be highlighted for their known function in increasing body mass index and intramyocellular lipid content^45^ as well as its genomic location within the “Tail fat deposition” QTL regions. Furthermore, seven Zel specific SNPs located in two genes (ETAA1 and PNO1) were observed within the candidate regions reported to link with fat-tail deposition in Ma et al. study^8^. Out of these SNPs, six were observed in ETAA1 gene in Zel, the latter was also determined in Yuan et al. study^6^ as an important candidate gene in fat-tail development. This gene also was reported in a comparative transcriptome analysis among three sheep breeds with different tail phenotypes^42^. Moreover, ETAA1 was differentially expressed in adipose tissue of sheep breeds with different tail types^46^ supporting its role in fat deposition. ETAA1 was also related to fat distribution in humans with African ancestry^47^ suggesting a similar contribution of this gene in fat-tail development in sheep breeds.

Mastrangelo et al.^48^ evaluated the signatures of positive selection for fat-tail in African and Eurasian sheep breeds and found 30 genomic regions, some of which harbored candidate genes associated with fat deposition. Our comparison revealed 18 (related to 10 genes) and 52 (related to 13 genes) SNPs that were located in these regions in Lori-Bakhtiari and Zel, respectively. Of the affected genes, CDS2 in Lori-Bakhtiari and PCDH9 in Zel were of greater interest, as they previously reported in the literature to be associated with fat deposition^46,49^. Both of the two SNPs of CDS2 were also reported in Yuan et al. study^6^. Also, PCDH9 with nine SNPs was located within a genomic region identified as ovine QTL regions for carcass fat percentage. These findings corroborated the role of these genes as important candidates for fat-tail development.

Additionally, two Zel specific SNPs were detected within the NRIP1 gene (also known as RIP140). This gene encodes a nuclear protein and is known as a regulator of lipid and glucose metabolism and fat deposition^50^. Xu et al. performed a GWAS study in fat- and thin-tailed sheep and reported NRIP1 as a regulator of lipid storage processes. Also, they reported STEAP4 and SLC29A4 as potential candidate genes^51^. In this study, genes containing Zel specific SNPs were included STEAP3 and SLC29A3, which are in the same gene family with STEAP4 and SLC29A4, respectively. Bone morphogenetic protein (BMP) family members are known for their ability to induce bone development^52^ with one family member, BMP2, involved specifically in fat-tail formation^5,6,48^. Higher fat deposition in the tail of sheep is partially attributed to higher expression of BMP2, which can induce stem cell differentiation into adipocytes^53^. We found other members of BMP family that included BMP6 with missense SNP in Lori-Bakhtiari and BMP1 with intronic SNP in Zel. Recently, BMP2 and BMP6 were proposed as new potent insulin-sensitizers in adipocytes^54^, which reinforce their potential function in fat-tail development. Zhang et al. performed a GWAS study in two lines of Hulun Buir sheep with different types of fat-tails and identified 33 SNPs located in 42 gene regions, 13 genes of which were considered as candidate genes for fat deposition^55^. Six of these 13 genes including one gene (FBF1 with one SNP) in Lori-Bakhtiari and five genes in Zel [MPST (four SNPs), MIA3 (15 SNPs including nine missenses), SETD7 (two SNPs), INTS9 (one SNP) and SFT2D2 (one SNP)] were identified in our study.

Fifty-seven and 141 genes harboring Lori-Bakhtiari and Zel specific SNPs were differentially expressed in fat-tail tissue of these breeds, respectively (Supplementary File S1). Functional enrichment analysis of these genes revealed significantly (FDR ≤ 0.05) enriched GO terms (including four BP and five MF) and no significant KEGG pathway. Several significant GO terms or KEGG pathways (P ≤ 0.05) were found to be related to fat metabolism including regulation of fat cell differentiation, positive regulation of lipid storage and PPAR signaling pathway (Supplementary File S5). The current results add further evidence to previous findings and confer important roles to the identified genes in fat-tail formation.

Considering that breed-specific SNPs might have been a consequence of selective pressures associated with fat metabolism traits, functional enrichment analysis was applied on the genes containing the breed-specific SNPs. This analysis resulted in identifying SNPs on the genic regions that were related to fatty acid metabolism in both breeds. Hence, our finding might be used to explore genomic regions and genes associated with fat-tail development. Most of fatty acid metabolism-related genes were grouped in fatty acid biosynthetic process, fatty acid oxidation, fatty acid metabolic and catabolic process, fatty acid beta-oxidation, fatty acid transport, fatty acid synthase activity, fatty acid biosynthesis, fatty acid degradation, fatty acid elongation, lipid biosynthetic process, regulation of lipid biosynthetic process, cellular lipid catabolic process, lipid homeostasis, PPAR signaling pathway and glycerolipid metabolism (Supplementary files S2 and S3). Among these GO terms and pathways, it is worthwhile to highlight some genes with a large number of SNPs in Zel including DGAT2 (50 SNPs), ACSL1 (32 SNPs), ADIPOQ (30 SNPs), ACACA (28 SNPs), ACSS2 (16 SNPs), ELOVL6 (16 SNPs), LPL (14 SNPs), ACLY (13 SNPs), ALDH7A1 (10 SNPs), CYP1B1 (nine SNPs), GPAM (nine SNPs), ECHS1 (eight SNPs), FADS1 (eight SNPs), FASN (eight SNPs), LSS (eight SNPs), SGPL1 (eight SNPs) and SCD (four SNPs). Also, some of the important fat metabolism-related genes in Lori-Bakhtiari were NUDT19 (five SNPs), ADIPOQ (two SNPs), CPT2 (two SNPs), ELOVL5 (two SNPs), HIBCH (two SNPs) and SORBS1 (two SNPs) (Supplementary File S1). Of these, four genes with the highest number of SNPs were DGAT2, ACSL1, ADIPOQ and ACACA. DGAT2 is a member of acyl-CoA diacylglycerol acyltransferase (DGAT) family that catalyzes the final step in the production of tricacylglycerols^56^. Our previous study showed that a silent SNP in DGAT1 can result in increased tail fat weight and backfat thickness in Lori-Bakhtiari compared to Zel^57^. Also, DGAT2 was reported to affect fat deposition in pig^58^. ACSL1 encodes an isozyme that converts free fatty acids into fatty acyl-CoA esters preparing them to form triacyglycerol. The crucial function of this gene in fatty acid biosynthesis, transport, storage and degradation in bovine has been documented^59,60^. Similar to our previous study on Lori-Bakhtiari^3^, ACSL1 was shown to be differentially expressed in subcutaneous fat of the Guangling (fat-tailed) and Han (thin-tailed)^61^ and in fat-tailed vs thin-tailed lines of Hulun Buir sheep^62^. ADIPOQ encodes production of adiponectin hormone that is closely related with regulation of glucose and fatty acid oxidation^63^. Importance of this gene in fat deposition was highlighted in a transcriptome-based study on sheep^11,61^. Moreover, association of genetic variants in ADIPOQ was documented with fat deposition and carcass traits in pigs^64^ as well as meat marbling in cattle^65^. ACACA encoded by a gene located in QTLs associated with fatness is the rate-limiting enzyme in de novo synthesis of long-chain fatty acids converting acetyl-CoA to malonyl-CoA^66^. A negative regulatory mechanism between an intergenic long non-coding RNA and ACACA was suggested for regulation of fat deposition in sheep in our previous work^10^. This gene has been also reported as a potential candidate for increased milk fat content in sheep^67,68^ and cattle^69^, intramuscular fat content in bovine skeletal muscle^70^ and fatty acid composition in pig meat^71^. ELOVL family is composed of seven fatty acid elongase isoforms (ELOVL1-7) involved in elongation of very-long-chain fatty acids. ELOVL6 specifically catalyzes the elongation of saturated and monounsaturated fatty acids, therefore, is very important in overall balance of fatty acid composition^72^. ELOVL genes (including ELOVL6 and ELOVL5) were differentially expressed in fat-tail tissue between Lanzhou (fat-tailed) and Tibetan (thin-tailed) sheep suggesting that they are among candidate genes contributing to shaping fat-tail phenotype^42^. Overall, these findings support that these genes are important regulators of fat deposition and play key roles in the morphological diversity between different sheep breeds.

Of the above highlighted genes, ACLY, CYP1B1, CPT2 were differentially expressed. For example, CPT2 that was located in QTLs associated with fatness was shown to act in partitioning a greater fat mass toward the tail than the viscera in Tan sheep^7^. CPT2 encodes carnitine palmitoyl transferase II that transfers palmitic acid from cytosol to the mitochondrial matrix^74^ and is a rate-limiting enzyme in long chain fatty acid β-oxidation^73^. Our findings along with evidence from previous studies suggest these genes as functional candidate genes contributing to fat deposition in tails of sheep.

A SNP in FABP4 gene was detected in Zel. FABP4 is a small cytosolic protein that specifically transports long chain fatty acids to the sites of storage^75^. This gene was candidated for being relevant to fat deposition in our previous studies^3,11^. Also, the role of this gene in determining the fat content has been highlighted in mouse^76^, cattle^77^ and pig^78^. Nine Zel specific SNPs were found in Homeobox genes including HOXD3 (six SNPs), HOXA10 (two SNPs) and HOXC12 (one SNP). Interestingly, in a recent study, HOX genes were suggested as novel candidates in regulating local fat distribution, which may result in diverse tail types in fat-tailed sheep breeds^7^.

Our previous study showed that most of the differentially expressed genes up-regulated in Zel compared to Lori-Bakhtiari were involved in fatty acid oxidation pathways such as PPAR signaling pathway^3^. Likewise, most of the breed-specific SNPs identified in this study were characterized in Zel. Moreover, GO terms and pathways related to fatty acid oxidation such as PPAR signaling pathway and fatty acid oxidation were significantly enriched in the genes containing Zel specific SNPs. These findings add further support to our previous study that the above-mentioned genes potentially stimulate fatty acid oxidation thereby preventing fat deposition in the tail of Zel.

## Conclusions

The comparison of two Iranian sheep breeds, substantially differing in tail phenotype (fat vs thin) increased the probability of identifying SNPs involved in shaping of the fat-tail. A large number of breed-specific SNPs belonging to different genes/genomic regions were identified that were located in the genic regions with known functions in fat-tail formation or fat metabolism. These SNPs were also mapped within QTL regions associated with fatness. Finding of the present study also suggested that the small tail of Zel can be related to the specific genes that stimulate fatty acid oxidation. Our results provided a foundation for further investigation of the genetic basis underlying fat-tail development in sheep. It should be noted that false positives can be of concern among the reported candidate genes, and further studies will be needed to unravel the complex mechanism of fat-tail development in different sheep breeds.

## Supplementary information

Supplementary file1

Supplementary file2

Supplementary file3

Supplementary file4

Supplementary file5
